# Advances in mechanical assessments of in vivo human lumbar spine tissues with noninvasive imaging techniques

**DOI:** 10.1038/s44385-026-00070-0

**Published:** 2026-02-18

**Authors:** Dawn M. Elliott, Harrah R. Newman, Mackenzie N. Conner, Curtis L. Johnson, Edward J. Vresilovic

**Affiliations:** Department of Biomedical Engineering, University of Delaware, Newark, DE USA

**Keywords:** Engineering, Health care, Medical research

## Abstract

Low back pain (LBP) is the leading cause of disability worldwide, yet clinical imaging remains largely limited to anatomical assessment, providing little insight into the spinal tissue mechanics underlying most idiopathic cases. This review highlights emerging noninvasive imaging technologies that enable in vivo quantification of intervertebral disc and spinal muscle mechanics, including radiography, ultrasound imaging, ultrasound elastography, magnetic resonance imaging, and magnetic resonance elastography. These approaches move beyond static morphology to capture spinal kinematics, load-dependent deformation, and tissue material properties under physiologically relevant conditions. Despite substantial technical progress, translation is hindered by inter-individual variability, limited symptomatic cohorts, and challenges in separating age-related changes from pathology. We discuss opportunities to accelerate clinical impact through development of normative mechanical datasets, dynamic and load-dependent imaging paradigms, and integration of imaging-derived mechanical biomarkers with computational modeling and machine learning. Together, these innovations position mechanics-based imaging to enable objective diagnosis, improved patient stratification, and mechanism-driven treatment of low back pain.

## Introduction

**Fig. 1:**
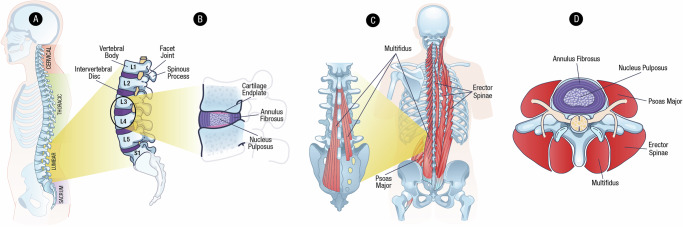
Schematic of spine anatomy. **A** Human sagittal section showing location of lumbar spine in yellow. **B** Lumbar spine showing intervertebral disc (including the annulus fibrosus, nucleus pulposus, and cartilage endplate), vertebral body, facet joint, and spinous process. **C** Human coronal section from the back showing the three major lumbar spine muscles groups: the erector spinae, multifidus, and psoas major. **D** Transverse section showing relative positions of the intervertebral disc (including the annulus fibrosus and nucleus pulposus) and the three major spinal muscle groups.

The spine is the central backbone of human motion, stability, and support. As a mechanical structure, it allows us to stand upright and bear weight, provides the motions needed to sit, walk, bend, and twist, and protects the spinal cord and nerve roots. These mechanical functions arise from the integration of spinal tissue subcomponents that each have distinct structures and material properties (Fig. 1). The spinal vertebrae are rigid bony elements which are the primary components providing structure and neural protection. The remaining structures allow stable motion and may be divided into passive and active restraints. The passive mechanical restraining tissues include the intervertebral disc between two vertebral bodies, two diarthrodial facet joints between the posterior vertebral elements, and at least 7 ligaments per spinal articulation. The dynamic restraints are the numerous inter-connecting spinal muscles (Fig. 1).

Low back pain (LBP) has been a leading cause of disability worldwide for several decades and remains one of the most common reasons for seeking medical care^1^. Outside of fracture, LBP rarely originates from the vertebrae themselves, leaving clinicians to manage symptoms attributed to dysfunction of the spine’s passive and active motion-restraining tissues. Despite extensive use of imaging, a specific pathological diagnosis is established in only ~10–15% of LBP patients, while the remaining 85–90% are classified as having nonspecific mechanical LBP^2–4^. In this large clinical population, the intervertebral disc and spinal muscles demonstrate structural alterations, making them leading candidates as sources of pain and disability. However, routine anatomical imaging of these tissues has limited ability to distinguish symptomatic from asymptomatic individuals or to guide treatment decisions. This diagnostic gap reflects a fundamental limitation of current clinical assessment: anatomy alone does not capture the mechanical dysfunction that likely drives symptoms, functional impairment, and disease progression in most patients. Noninvasive, in vivo evaluation of disc and muscle mechanics has the potential to transform clinical care by enabling objective diagnosis, improving patient stratification, and supporting more precise, mechanism-based prevention and treatment strategies. Although conventional imaging readily characterizes disc and muscle morphology^5,6^, quantifying their mechanical function in the living human spine remains challenging. This review highlights recent advances in noninvasive imaging techniques designed to assess spinal tissue mechanics, with a focus on measurements of intervertebral disc and spinal muscle function.

### Structure and mechanical function of spinal tissues – intervertebral disc and spinal muscles

The intervertebral disc is composed of three main components (Fig. 1B, D): the central nucleus pulposus (NP), the surrounding annulus fibrosus (AF), and the cartilaginous endplates^7^. The NP is a gelatinous, proteoglycan-rich core that generates osmotic swelling pressure, transmitting outward forces to the AF under axial loading. The AF consists of concentric collagen lamellae with fibers arranged in alternating orientations, which resist multidirectional loads and provide high tensile stiffness, enabling spinal flexibility while serving as a passive stabilizer. The cartilaginous endplates anchor the NP and inner AF to adjacent vertebral bodies, serving as a semipermeable barrier for fluid exchange and nutrient transport.

The lumbar spine is also supported by key muscle groups: the erector spinae, multifidus, and psoas^8^ (Fig. 1C). The erector spinae consist of three long and powerful muscle groups that run parallel to the spinal column. The erector spinae are responsible for extension and lateral bending, and by resisting flexion, allow upright posture and controlled movement. The multifidus group consists of short, deep muscles attaching to each vertebra along both sides of the spine, providing segmental stability, fine motion control, and resistance to shear forces. The psoas connects the lumbar vertebrae to the femur, functioning as a hip flexor while also transmitting compressive and shear forces across the lumbar spine.

### Successes and challenges in quantifying in vivo spine mechanics

Imaging modalities can readily identify tissue structure^5,6^, but they do not directly capture mechanical function. Quantifying mechanical function requires evaluating tissue responses to controlled loading, since stiffness and related properties are defined by the *change* in force or stress relative to the *change* in displacement or strain^7^. For example, measuring how much load increases as a tissue elongates, or how displacement changes under increasing compression, reveals the fundamental material characteristics of a tissue. Accurate measurement requires that responses be captured without disrupting the natural loading environment. In vivo, this presents major challenges: spinal tissues are located deep within the body, surrounded by bony structures, internal organs, and fat, and consist of numerous interconnected components with distinct material properties and overlapping functions. Variability across individuals, including differences in age, sex, and pathology, further complicates interpretation of measurements. These factors make isolating and quantifying the contribution of individual spinal tissues difficult.

### Semi-invasive assessments

Semi-invasive assessments, such as electromyography (EMG)^9^ and pressure transducers (with or without injection of contrast)^10–12^ have advanced in vivo measures of spinal tissue mechanics but have limitations that curtail routine use in humans. Fine-wire or needle EMG provide muscle-specific recordings from deep muscles like the multifidus and psoas, but causes discomfort, needle-induced strength changes, and signal contamination from adjacent muscles^13–15^. Surface EMG offers a noninvasive alternative for assessing global activity in large superficial muscles, though it is limited by crosstalk and an inability to measure absolute force. Needle-mounted pressure transducers and stress profilometry have been used in the research setting to measure in vivo disc pressure across postures and loading conditions^10–12,16,17^. Discography, which involves fluoroscopically guided needle insertion into the disc and injection of contrast-enhanced fluid, can be used clinically to localize painful discs^18,19^. Discography offers the opportunity to quantify nucleus pulposus (NP) pressure, a key metric in disc mechanics, and thereby provides insight into disc mechanics in both asymptomatic and LBP populations^20,21^. Moreover, discography often provides visualization on structural damage in the annulus fibrosus and cartilaginous endplate which may not be visualized in other imaging modalities and may have implications for altered tissue mechanical loading as part of the degenerative cascade^22^. However, insertion of needle-mounted pressure transducers and discography needles carry risks of disc injury and accelerating degeneration which has restricted their use diagnostically and in human studies^18,23,24^. Yet these approaches remain important in cadaveric and animal research for establishing robust baseline data that inform predictive modeling and ex vivo biomechanical testing^25–27^.

### Modeling

Numerical and computational models of the lumbar spine and intervertebral discs have provided important mechanistic insight by isolating the contributions of individual tissues and structural components across a broad range of loading conditions, magnitudes, and rates, in healthy, degenerate, and pathological states^28–30^. The predictive capability of these models depends critically on accurate representation of anatomy, constitutive material behavior, tissue interactions, boundary conditions, and loading. Anatomical geometry can be reliably obtained using established imaging modalities such as radiography^31^ and MRI^32^. In contrast, specification of physiologically relevant material properties remains a major limitation. Experimental characterization of individual tissue constituents typically requires excision from the native mechanical environment, while in vivo assessment of soft tissue constitutive behavior is constrained by anatomical complexity and current imaging capabilities. Consequently, models often rely on simplified or literature-derived material properties, introducing uncertainty and limiting validation. Experimental validation is further challenged by the scarcity of in vivo data describing load-dependent tissue deformation and kinematics. Noninvasive imaging techniques capable of quantifying spinal kinematics and deformation under physiological loading provide an opportunity to inform model inputs, constrain parameter estimation, and support validation. When integrated with imaging-derived measures of in vivo behavior, computational models can serve as powerful tools for investigating spinal pathology and enabling subject-specific predictions^33,34^.

### Low back pain

Low back pain (LBP) is the leading cause of disability worldwide and its prevalence is increasing. LBP affects nearly 620 million people globally, and chronic LBP, pain lasting more than 3 months, affects 8% of US adults^35,36^. LBP also accounts for more US healthcare expenditures than any other musculoskeletal condition^36,37^. The majority of LBP is idiopathic^3^, with no clear structural, anatomical, or pathological cause detectable on routine imaging. Moreover, most idiopathic LBP is mechanical in nature^38–41^. That is, pain is triggered by activity or modulated by posture and load. Examples of mechanical LBP include pain exacerbated by standing, sitting, lifting, or bending. In general, the lack of pathoanatomical cause and insufficiency of static imaging has led to the lack of successful targeted diagnoses and treatments^6^. Because mechanical LBP indicates altered biomechanics and tissue function, advances of in vivo assessments of spinal tissue mechanics may improve LBP diagnosis and treatment.

### Aging and degeneration

Discs and spinal muscles undergo major degenerative alterations with normal aging. Disc degeneration is characterized by reduced hydration and structural integrity. X-ray and MRI are both commonly used to assess loss of disc height and evaluate the degeneration severity with qualitative grading schemes^42,43^ which can aid in clinical assessment and isolation of potentially pathological disc levels. MRI also enables quantitative measures such as T1rho, T2, and T2* relaxation times, which correlate with tissue composition^44–47^. Incidental findings of disc degeneration occur even in young asymptomatic populations and increase with age, independent of pain^6,48–50^. Similarly, paraspinal muscle degeneration, including atrophy and fatty infiltration, is typically evaluated from axial MRI using grading schemes or quantitative fat fraction measures^51–53^, and also increases with age in asymptomatic populations^54,55^. These age-related structural changes impair spinal motion and stability^56,57^, yet the high prevalence of degeneration in asymptomatic individuals complicates interpretation^6^. Current imaging cannot reliably distinguish between expected aging and pathology, and correlations between degenerative changes and LBP remain weak or absent^58–61^. This diagnostic ambiguity underscores the need for functional and mechanistic assessments of spinal tissues.

Quantifying the mechanical contribution of the discs and muscles to whole-body motion is difficult, but can be inferred using motion capture systems or wearable technologies^62,63^. Computational musculoskeletal models, such as OpenSim, can estimate joint forces, muscle activations, and segmental loading^64^ and finite element analysis of spinal segments, such as in FEBio^34,65^, can estimate regional stresses and strains under physiological loading conditions. Isolating each tissue’s contribution to spinal motion or segmental mechanics using these tools, however, requires accurate tissue material properties and boundary conditions, which are difficult to determine in vivo. Quantification of expected contributions from each tissue is advantageous, as in pathological cases it can aid in identifying abnormal loading and isolation of unexpected mechanical contributions can help provide specific diagnosis which can enable a more targeted treatment or rehabilitation approach.

Advances in non-invasive imaging now enable visualization and quantification of tissue mechanics under physiological loading. This review focuses on non-invasive approaches—radiography, ultrasound imaging, ultrasound elastography, magnetic resonance imaging, and magnetic resonance elastography—focusing on the early seminal work and recent advances for each modality in measurement of disc and muscle tissue mechanics. Additionally, we provide the authors’ assessment of the strengths, limitations, and future outlook for advancing in vivo mechanistic understanding of spinal mechanics for each modality.

## Radiography

**Fig. 2:**
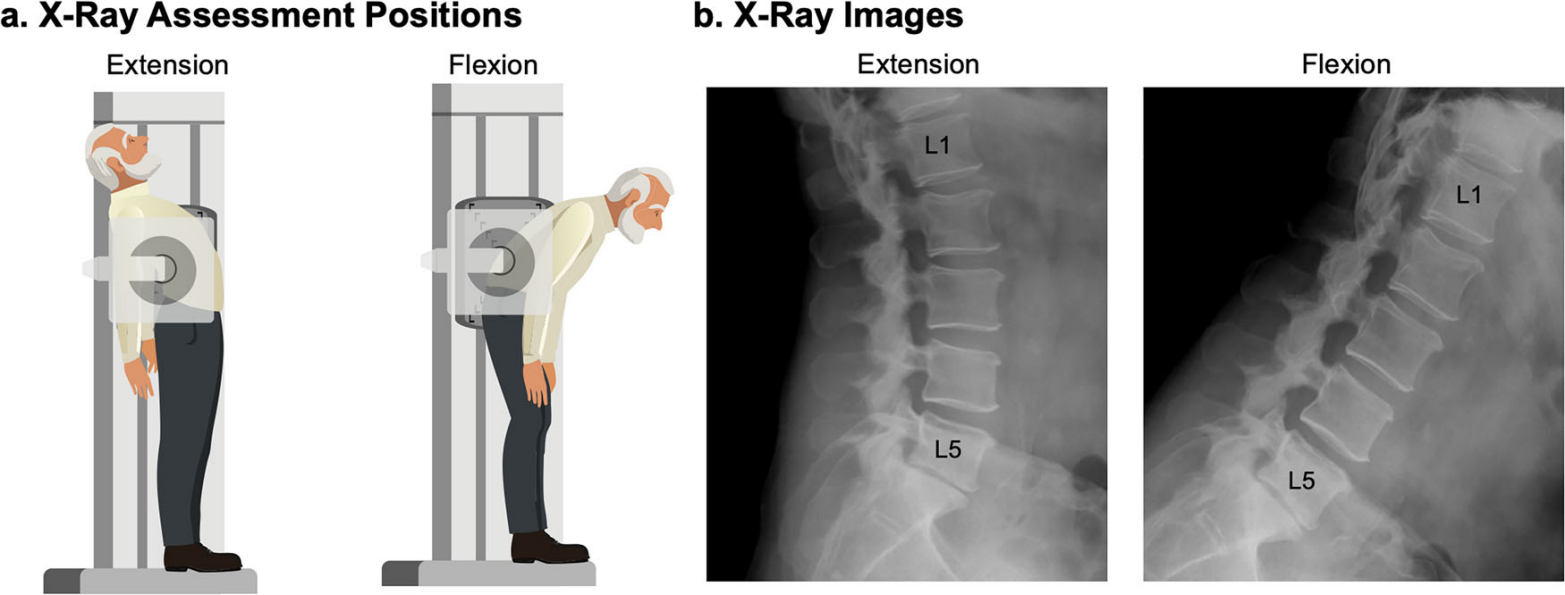
Radiography. Disc mechanics can be indirectly assessed from repeated radiographs under **a** postural loading, for example extension and flexion. Disc boundaries are measured by their adjacent bony locations to calculate change in disc height, wedge angle, or translation. **b** Lumbar spine extension and flexion X-ray images.

Radiography (Fig. 2) uses X-rays, high-energy electromagnetic waves that penetrate tissues, to generate contrast based on differential absorption. Dense structures such as bone absorb more radiation and appear bright, whereas low-density tissues appear darker. Early applications in the 20th century relied on planar two-dimensional projections^66^, followed in the 1940s by the introduction of radiopaque contrast agents, which enabled visualization of soft tissues and pathologies such as disc herniations^67^, and biplanar radiography allowed orthogonal views^68^. The development of computed tomography (CT) in the 1970s marked a major advance, using rotating X-ray sources and computational reconstruction to generate volumetric three-dimensional images^69,70^. More recently, EOS spinal imaging has been introduced, using simultaneous biplanar acquisition with slot-scanning technology to provide ultra–low-dose, three-dimensional imaging of the spine. This approach enables assessment of spinal alignment in upright, weight-bearing postures, capturing functional alignment that cannot be evaluated with conventional supine imaging^71,72^. Radiography is a primary tool for assessing spinal morphology and pathology, particularly those involving bone, including vertebral fracture, stenosis, spondylolisthesis, and scoliosis. Decades of work have established normative data for regional curvature and range of motion for the spine from static images in one or multiple positions^73–76^. As a fundamental diagnostic modality for more than a century, its strengths are rapid acquisition, accessibility, and clinical utility in both diagnostic and intra-operative applications. Nonetheless, ionizing radiation presents risks of DNA damage and carcinogenesis, necessitating judicious use, particularly in younger populations. Accordingly, the benefits of radiographic assessment must be weighed against the risks of cumulative exposure, and imaging protocols should prioritize minimizing unnecessary scans and carefully planning follow-up studies^5,77^.

### Radiography – intervertebral disc mechanics

Disc function can be indirectly assessed from repeated radiographic imaging under postural loading and quantification of vertebral translations and rotations across postures (Fig. 2). 2D X-ray images collapse the 3D spine into a single plane of view that can be sufficient for evaluating overall spine structure and curvature, such as in the assessment of scoliosis/lordosis, while CT imaging can provide volumetric information useful for distinguishing 3D translations and rotations. Flexion typically induces a reduction in wedge angle changes up to <10° and anterior vertebral translation of 1–3 mm, while extension, limited by the posterior facet joints, produces a smaller increase in wedge angle <5° and posterior translation of <1 mm^73,74,76^. Translations and rotations during flexion and extension can distinguish healthy from pathologic populations. The distribution of translations my also provide diagnostic information: healthy individuals exhibit greater contributions from lower lumbar levels in standing and extension, whereas motion in patients with LBP shift to upper lumbar levels, likely reflecting compensatory strategies involving discs and muscles^78^. Radiography is also useful in functional loading studies. Even modest tasks, such as lifting 10 lb, induce intervertebral shear (~5 mm) at all lumbar levels, with nearly equal contributions from L2 to L5 and reduced motion at L5–S1, reflecting pelvic–sacral anatomical constraints^79^. Implanted markers further extend radiography to kinematic and strain analysis in both experimental and clinical contexts^68,80^. Despite its limitations, radiography remains central in scoliosis assessment, where it is used to monitor curve progression and guide intervention^81^, and in spondylolisthesis, where spinal instability is quantified by the anterior displacement of one vertebra relative to the adjacent vertebra in standing radiographs^82,83^.

### Radiography – spinal muscle mechanics

Radiographic assessment of spinal muscles has focused on cross-sectional area as a marker of atrophy and tissue density as an indicator of fatty infiltration. Both are indirect measures of mechanical function, and the poor soft-tissue contrast of radiography limits its ability to capture more than partial aspects of muscle function.

### Radiography – outlook

Radiography will continue to play a central role in evaluating bony abnormalities because of its accessibility and diagnostic value. However, its limited ability to assess soft tissues and the risks associated with repeated radiation exposure constrain its use in functional studies. Emerging advances that may extend its applications to spinal function include low-dose imaging protocols^71,72,84–86^, enhanced image processing and motion analysis^73,74,76^, and integration with complementary modalities such as MRI or ultrasound.

## Ultrasound imaging

**Fig. 3:**
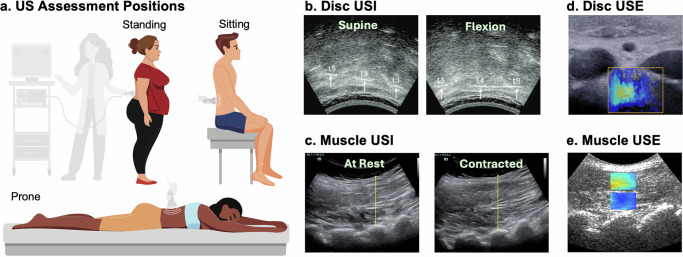
Ultrasound imaging (USI) and Ultrasound elastography (USE). USI acquired at **a** different positions, which are often combined with activity such as arm or leg raising or torso bending, can be used to estimate disc kinematics and muscle activation as contraction-induced thickness change. **b** Sagittal disc USI showing L3–L5 spinous process locations in supine and flexion, adapted from ref. ^97^. **c** USI of lumbar multifidus muscle thickness at rest and during contraction to measure change in thickness, adapted from ref. ^238^. USE extends USI to provide mechanical properties rather than just anatomical information. Advanced technology to induce vibration or shear waves are required (not shown). **d** USE of an adolescent disc at the L3–L4 annulus fibrosus, where warmer color indicates greater shear wave speed, adapted from ref. ^127^. **e** USE of L4–L5 multifidus muscle superficial (top ROI) and deeper (bottom ROI) layer, where warmer color indicates greater stiffness, adapted from ref. ^135^.

Ultrasound imaging (USI) (Fig. 3) is a noninvasive modality that visualizes internal tissues by transmitting high-frequency sound waves and detecting their echoes with a transducer^87^. Ultrasound waves are generated by a piezoelectric crystal within the transducer and are reflected at tissue interfaces according to differences in acoustic impedance; consequentially interfaces with greater differences in density produce stronger echoes, which appear brighter on the image. The most widely used application, brightness mode (B-mode), generates two-dimensional grayscale images, with image planes determined by probe positioning. Medical ultrasound was first introduced in the 1940s for detecting brain tumors and subsequently expanded across clinical applications, with musculoskeletal imaging emerging in 1958^88^.

Ultrasound offers several advantages. It is portable, low cost, noninvasive, and radiation-free, making it particularly suitable for repeated use in sensitive populations such as children and patients undergoing rehabilitation^87,89^. Its portability also enables dynamic imaging of postures and movement, supporting both clinical practice and research applications. Yet ultrasound has important limitations, particularly signal attenuation, which limits visualization of deeper structures, and challenges in obtaining high quality and consistent images due to operator dependence, steep learning curves, and variable protocols^89–92^.

### USI – intervertebral disc mechanics

Ultrasound imaging was first applied to the spine in the 1980s to visualize the epidural space^93^. By 2000, disc structure could be imaged via a posterolateral approach, with identifiable features between T11 and L3^94^. Since then, spine USI has been primarily for image-guided procedures, where accuracy is improved with real-time visualization for epidural and facet joint injections. Additionally, USI has been employed to measure Cobb angle, showing potential as a radiation-free screening and monitoring tool for scoliosis^95,96^.

USI has been explored for assessing spinal kinematics by measuring displacement between spinous processes during motion, taking advantage of the unique handheld nature of the USI equipment (Fig. 3). Comparisons with MRI have demonstrated that, while USI can reliably detect interspinous displacement (Fig. 3b), it has a lower sensitivity than MRI^97^. Subsequent kinematic studies during flexion or rotation report good to excellent reliability for interspinous displacement in healthy populations^98^ and during lumbar rotation, demonstrating that most axial twist is localized to L2–L3 and L5–S1^99^. USI has also been used to evaluate lumbar mobility following mobilization treatments, detecting increases in flexion and extension following intervention^100^. These studies demonstrate potential utility; however, despite these advances, USI-based biomechanical assessment remains largely limited to feasibility studies, and its role in disc pathology and functional spinal biomechanics is still underdeveloped.

### USI – spinal muscle mechanics

Ultrasound imaging of lumbar muscles was validated against MRI in the 1990s, with cross-sectional area measurements showing no significant differences between modalities^101^. This validation supported the development of normative data for multifidus thickness and encouraged use of USI to assess muscle activation through contraction-induced thickness changes (Fig. 3c)^102^. Applications have since expanded to the psoas and erector spinae, where USI has been used to quantify thickness and area changes during functional tasks (Fig. 3)^103–106^. Thickness changes correlate well with electromyography (EMG) in asymptomatic populations under controlled conditions^107–109^, suggesting potential as a proxy for muscle activity quantification. However, findings are less consistent in symptomatic populations, where thickness change may not be a reliable surrogate^110,111^. USI has been widely applied to study lumbar muscles in individuals with LBP, particularly the multifidus, with altered activity reported in conditions such as disc herniation and sacroiliac joint pain^112,113^. However, results for idiopathic LBP are variable: some studies report reduced activation^105,114,115^, whereas others find no significant differences compared with controls^116,117^. This heterogeneity highlights the need for caution in interpreting muscle mechanics from USI-derived measures in clinical populations.

### USI – outlook

USI is uniquely portable, radiation-free and can be used to evaluate spinal kinematics and lumbar muscle function longitudinally; however, its broader implementation remains constrained by methodological and clinical limitations. Acoustic attenuation restricts penetration depth, limiting visualization of deeper structures such as the lumbar intervertebral discs and psoas muscle. Variability in protocols, such as contraction strategies and patient positioning, reduces reproducibility and complicates comparisons across studies. Additionally, image quality and interpretation are highly operator dependent, with a steep learning curve for reliable acquisition. Technological advances such as high-resolution probes, three-dimensional ultrasound, and automated segmentation hold promise for enhancing functional assessments, particularly of deeper structures. Integration with complementary modalities, including MRI, EMG, and motion analysis, may further provide multimodal insight into spinal mechanics and neuromuscular control. Reliable validation and demonstration that USI parameters provide clinically meaningful information for pathology and treatment response are needed before widespread adoption. Finally, future work should emphasize standardized acquisition protocols and operator training to improve reproducibility across populations and studies. If these challenges are addressed, USI may grow as a valuable, radiation-free modality for functional assessment and longitudinal monitoring of the disc and spinal musculature in both research and clinical settings.

## Ultrasound elastography

Ultrasound elastography (USE) extends conventional ultrasound by quantifying tissue stiffness under mechanical perturbation, providing functional rather than purely anatomical information^118–120^. Perturbations may be generated by manual probe compression, externally applied vibrations, or focused ultrasound pulses that create internal acoustic waves. USE encompasses two main approaches: strain elastography, which measures tissue deformation under compression, and shear wave elastography (SWE), where shear wave speed reflects stiffness, with higher speeds indicating greater stiffness^121^. First introduced in 1991, when ultrasound was shown to detect tissue deformation under compression^122^, USE was soon applied to skeletal muscle, with early studies demonstrating that vibration-induced shear waves detected stiffness increases during quadriceps contraction^123^. USE offers quantitative, noninvasive, portable, and radiation-free assessment of spinal tissues, enabling repeated evaluation of stiffness.

### USE – intervertebral disc mechanics

The first application of shear wave elastography (SWE) to intervertebral disc was in the cervical spine^124^. Subsequent lumbar studies demonstrated that SWE can quantify shear wave speed in the anterior annulus fibrosus (AF) (Fig. 3d), with values of ~3 m/s in healthy children and ~4 m/s in adults, showing good repeatability^125,126^. Clinical work has largely focused on adolescent scoliosis, where SWE has proven sensitive to pathological changes. While AF stiffness is consistently higher in L3-S1 scoliotic discs (3.5–4.0 m/s) compared to asymptomatic controls (3.1 m/s), there is not consistently a correlation between AF stiffness and scoliotic curve severity^127,128^. Long-term follow-up in neuromuscular scoliosis treated with fusionless fixation demonstrated persistently elevated AF stiffness compared to healthy peers (9.9 vs. 7.5 m/s)^129^. Together, these findings highlight the ability of SWE to detect stiffness changes associated with pathology and treatment. However, disc USE remains technically constrained: measurements are limited to the anterior AF of the lower lumbar spine (L3–S1), with the thoracic cage restricting access above L3. Feasibility is further reduced in high-BMI populations, as higher body mass impedes signal acquisition. Thus, while disc USE demonstrates clinical promise, its current scope is confined to a narrow anatomical region and specific patient populations.

### USE – spinal muscle mechanics

USE has been more extensively applied to spinal muscles than to discs. Strain elastography was first reported in 2012^130^, followed by SWE^131^, which has since become the preferred method. SWE values are a measure of tissue stiffness, and this stiffness correlates with muscle force production, thus reflecting muscle activity^132,133^, providing reliable assessments of activity in both superficial (erector spinae) and deep (multifidus) lumbar muscle (Fig. 3e)^118,131,134–136^. In healthy individuals, USE has been used to examine stiffness responses to posture, fatigue, and physical activity level, where stiffness increases with flexion, lateral bending, and contraction^137,138^ and decreases with fatigue^139,140^.

Clinical studies consistently demonstrate altered stiffness profiles in populations with LBP, with increased resting stiffness of the multifidus and erector spinae, accompanied by reduced contraction ratio during activity, compared with asymptomatic controls^113,141–143^. Further, SWE detects reduced multifidus stiffness on the affected side in patients with unilateral disc herniation^144^. Together, these findings highlight the potential of USE to capture alterations in spinal muscle mechanics associated with pathology. USE has also been used to evaluate treatment effects, with early promising results in small population studies. For example, in LBP participants, strain elastography demonstrated reductions in multifidus stiffness after capacitive and resistive electric transfer therapy^145^. Similarly, SWE has shown decreased erector spinae stiffness following dry needling^146^ and reductions in multifidus stiffness after radiofrequency neurotomy and lumbar fusion, particularly in active postures^147^. Overall, USE is sensitive to measure muscle function, but its broader utility is constrained by protocol variability, small sample sizes, and reduced reliability for deeper muscles such as the psoas.

### USE – outlook

USE is a promising technique for assessing both passive tissue mechanical properties and functional responses to activity in intervertebral discs and spinal muscles. However, across both tissues, reported stiffness values vary due to methodological differences, and few studies have investigated symptomatic subject populations. Furthermore, although posture-dependent changes have been described, most assessments are performed in static prone or supine positions, which may not capture physiologically relevant loading. Development of dynamic, upright, or weight-bearing protocols could improve functional assessment by capturing posture- or load-dependent responses not evident in supine imaging. Given the portability of ultrasound and the ease with which it can be used in a variety of positions, it provides unique opportunities to monitor spinal tissue mechanics during exercises or therapies and these measurements could influence treatment plans and guide rehabilitation strategies to improve patient outcomes.

Future priorities should include expanding feasibility across spinal levels and tissue regions. Technical advances, including high-resolution probes and improved penetration depth, will be necessary to extend applications to deeper structures such as the posterior AF, nucleus pulposus, and psoas. Other priorities are to establish normative reference data stratified by age, sex, BMI, and validating stiffness as a biomarker of pathology and treatment. Normalization strategies, such as referencing disc stiffness against another soft tissue site, may help reduce inter-device variability. Finally, integration with MRI, EMG, and motion analysis will provide richer, multimodal insight into spine function. With these developments, USE has the potential to evolve into a clinically valuable tool for monitoring spinal tissue mechanics and guiding rehabilitation and treatment strategies.

## Magnetic resonance imaging (MRI)

**Fig. 4:**
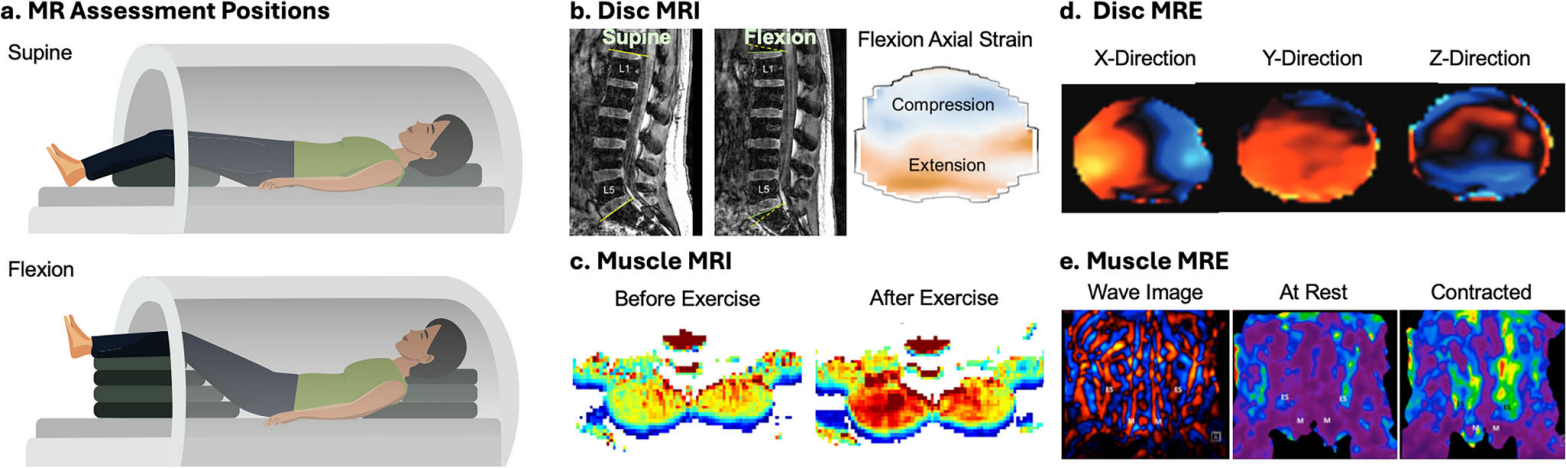
Magnetic resonance imaging (MRI) and Magnetic Resonance Elastography (MRE). MRI acquired under **a** different positions can be used to measure disc deformation and strain; and MRI before and after exercise can be used to estimate muscle activation through area and signal intensity change. **b** MRI of lumbar spine shown as midsagittal section in supine and flexion. Images are registered to calculate the axial strain in flexion relative to supine, shown in an axial section, adapted from ref. ^156^. **c** MRI T2 map (related to hydration) of paraspinal muscles before and after exercise showing increased T2 values after exercise, due to blood perfusion into the muscle, adapted from ref. ^239^. MRE extends MRI to provide mechanical properties rather than just anatomical information. Additional technology to induce vibration or shear waves are required (not shown). **d** In-plane and through-plane phase encoding MRE showing wave propagation through the disc, reproduced from ref. ^206^. **e** MRE of L2-L5 coronal view through the center of the muscles’ cross-section, showing the wave image and the shear modulus map while at rest and while contracting, where warmer colors indicate stiffer tissues; erector spinae (ES) and multifidus (M) both become stiffer with contraction, adapted from ref. ^225^.

Magnetic resonance imaging (MRI) (Fig. 4) relies on an external magnetic field to align protons within tissues. Radiofrequency (RF) pulses perturb this alignment, and the energy released during realignment is detected to generate images. Because the rate and magnitude of energy release depend on tissue composition, MRI provides excellent soft tissue contrast and enables clear differentiation between spinal structures. The first human body MRI was performed in the 1970s^148^. Since its early spinal applications in the 1980s, MRI has steadily expanded as a key imaging modality for studying the spine, capturing structural anatomy and tissue changes associated with aging and degeneration, and is used clinically to detect abnormalities such as disc herniation, spinal stenosis, spondylolisthesis, and tumors^5,149^. Anatomic imaging is not effective in identifying the source of idiopathic mechanical LBP, motivating recent efforts to use MRI under loading conditions to assess disc and muscle mechanical function.

### MRI – intervertebral disc mechanics

MRI has been adapted to evaluate disc mechanics by mathematically registering baseline scans to those obtained during mechanical loading or posture changes (Fig. 4), such as diurnal compression (assessment of changes between morning and evening), acute compression (induced by external load or exercise), or postural changes (induced by flexion, extension, lateral bending, or torsion). Disc mechanics have also been assessed using an upright MRI scanner, which permits more physiological loading in the standing and sitting postures^149–151^. Across postures and loading cases, a variety of methods have evolved for quantifying disc strain^152–157^, which is often evaluated as it quantifies the discs’ mechanical behavior while normalizing to the discs’ in vivo resting state. MRI further enables quantification of relaxation times such as T1rho, T2, and T2*. Though relaxation times do not directly evaluate disc mechanics, they are highly correlated with tissue composition, water content, and disc pressurization^20,44–47,158^, all of which influence the disc’s mechanical behavior. Relaxation times are generally greater in the upper lumbar levels than the lower lumbar levels^159^, and are regionally-dependent^159,160^, with the greatest relaxation times in the NP. Further, MRI relaxation times are consistently reduced in painful discs^20,58^ and degenerate discs^44,161,162^ compared to healthy controls and/or non-painful discs.

Diurnal loading studies show that daily compressive loading reduces disc height and hydration, particularly in young, healthy discs^156,163–165^. These effects are diminished in older or degenerated discs which have lower baseline hydration^161,164,166^. In young, asymptomatic subjects, compressive strain between morning and evening is typically 5–8%^156,159^. Some studies report level- or region-specific differences, with greater posterior strain at lower lumbar levels, while others found no variation. Diurnal loading induces minimal changes in wedge angle and shear displacement^156^. Diurnal changes in relaxation time, which imply loss of water content due to daily compressive load, are measurable in young, healthy discs^156,165–167^, but not consistently present in older, degenerated discs^166^. Diurnal change in relaxation times has been weakly correlated with diurnal disc strain and diurnal perimeter change^156,159^. Diurnal loading assessment requires MRI scans in both morning and evening, limiting practicality compared with single session loading protocols.

Acute compression applied with external loading devices produce greater deformation at the lower lumbar levels than diurnal loading^168,169^. Correlations between in vivo strain and degeneration are generally weak^168^. Unlike diurnal loading, externally imposed compression produces larger strains in older subjects, likely reflecting reduced disc stiffness that alters acute mechanical responses^155^. This is consistent with ex vivo mechanical testing, where intact motion segments tend to have reduced modulus with increasing degeneration, despite testing of isolated AF and NP tissues, which having increased stiffness with increasing degeneration^170–174^. In addition, treadmill exercise induces compressive strain in the lumbar discs^152^. Relaxation times do not significantly change with acute compressive loading^175^.

Postural loading, where subjects are placed in flexion, extension, lateral bending, or axial torsion postures, can be achieved within the MRI bore using bolsters to hold each position during scanning (Fig. 4b). Of these, flexion and extension have been studied most extensively. Flexion produces posterior migration of the nucleus pulposus, while extension results in anterior migration^176,177^. Wedge angles decrease during flexion and increase with extension^156,177^. Both postures generate regional AF strains: flexion causes anterior compression and posterior tension, while extension induces anterior tension and posterior compression^156,178,179^. Lateral bending and torsion have been less frequently investigated with small subject numbers^178,180,181^, though they may help characterize asymmetric loading such as in scoliosis.

Conventional MRI is typically performed in a 1.5 or 3.0 Tesla horizontal bore scanner with the subject supine on a table supported by pillows or bolsters (Fig. 4a). This setup is limited by the absence of axial gravitational load and the restricted range of motion within the bore. To address these limitations, upright MRI has been used to evaluate spine loading under normal gravitational conditions and with greater motion^150^. However, upright MRI generally operates at lower field strength (~0.5 T), which reduces resolution and available outcome measures compared to conventional MRI^182^. Despite this drawback, gravitational loading strongly influences spinal mechanics: flexion in upright posture produces distinct wedge angle distributions correlated with facet joint orientation^183^, whereas supine flexion in conventional MRI yields a more uniform loading pattern across the lumbar spine^156,177^.

MRI with loading has provided important insights into disc mechanics, yet several limitations remain. Biological variability for the disc mechanical response within and between subjects complicates interpretation and makes it difficult to establish normative values, even in young, asymptomatic populations^156,159,179,184^. This is particularly notable at L5–S1, the most anatomically variable disc in the lumbar spine, which often deviates from trends seen at other lumbar levels^156^. Although outcomes can be normalized by age or disc level, inconsistent trends and inconclusive findings persist across studies. Clinical application is further limited by few studies in symptomatic cohorts and small sample sizes that preclude generalizable conclusions. Technical variability across studies also hinders comparison, including differences in image intensity (e.g., from T1 and T2 weighting, etc.), field strength (0.5–3.0 T), in-plane resolution, and slice thickness. Computationally, quantifying mechanics requires repeated imaging with segmentation and registration; manual methods are labor-intensive and automated segmentation approaches are developing^33,185,186^. Finally, registration approaches for strain estimation, while showing promising outcomes, remain under active development in vivo and in cadaver experiments, and still require validation^156,169,187,188^.

### MRI – spinal muscle mechanics

MRI indirectly detects muscle activation through exercise-induced fluid influx, which increases muscle size and elevates signal intensity (Fig. 4c)^189^. Increased blood perfusion into the muscle has been measured by arterial spin labeling (ASL) techniques, but have shown minimal changes with pathology^190,191^. Flexion/extension tasks increase the cross-sectional area and signal intensity of the multifidus and erector spinae, confirming their recruitment during activity^192–194^. MRI has also been used to assess muscle function in pathological states, where decreased muscle area and reduced signal intensity have been interpreted as less active muscle in post-surgical LBP patients^193^. However, findings across studies remain inconsistent, sample sizes of symptomatic subjects are small, and MRI signal intensity should be regarded only as an indirect marker of spinal muscle activity.

### MRI – outlook

There remain meaningful opportunities for advancing MRI-based evaluation of disc and muscle mechanics. To date, most studies have focused on young, healthy populations, moving forward greater attention is needed to characterize age-related changes and to distinguish these from pathological alterations associated with LBP. Establishing such distinctions is essential for identifying mechanisms that separate pain-inducing pathology from expected aging, and to enable identification of impactful outcomes for therapeutic purposes. Upright MRI offers potential for assessing posture-dependent pain and asymmetric loading, but its clinical value remains limited by low field strength and poor resolution, which must be overcome before it can become a widely used tool. Finally, although anatomical studies have shown correlations between disc degeneration, muscle atrophy, and fatty infiltration^59,195^, the mechanical interplay between discs and muscles remains poorly understood. Paired investigations are needed to clarify the functional relationships between these adjacent tissues and to determine how they influence one another with aging and in LBP.

## Magnetic resonance elastography

Magnetic resonance elastography (MRE) is an MRI-based technique for quantifying soft tissue mechanical properties^196,197^. In MRE, harmonic mechanical vibrations are applied to generate tissue deformations, including propagating shear waves within the region of interest. Specialized pulse sequences incorporate motion-encoding gradients to map wave-induced displacements into the MR signal phase, and repeated sampling across the vibration cycle produces time-resolved images of shear wave propagation. Shear wave behavior reflects tissue material properties: stiffer tissues transmit waves faster, producing longer wavelengths, whereas viscous tissues dampen wave amplitudes. These dynamics are expressed as the complex shear modulus, in which the real component (storage modulus) describes elasticity, and the imaginary component (loss modulus) describes viscosity^198^. Studies report shear wave speed (m/s) or calculate shear modulus (kPa, often called shear stiffness) from frequency analysis, with higher values indicating greater stiffness^199,200^. These measures generate quantitative, spatially-resolved maps of tissue mechanical properties, positioning MRE as a powerful tool for noninvasive biomechanical assessment.

MRE, initially developed in 1995^196^, has since become a standard tool for diagnosing and staging liver fibrosis and chronic disease due to its high sensitivity and specificity^201^. Applications have expanded to the breast, brain, and other organs, where sensitivity to pathological changes and treatment responses in tissue mechanics offers diagnostic value^202–204^. The core components of each MRE application are the same: vibration of tissue to generate shear deformation or waves, imaging of the resulting wave displacements via specialized phase-contrast MRI sequences, and estimation of mechanical properties via computational “inversion algorithm”^198,204^. In practice, these components are tailored and optimized for each tissue of interest to generate reliable and useful outcome measures by accounting for differences in tissue anatomy, geometry, and mechanical behavior, which is critical in application to spinal tissues.

### MRE – intervertebral disc mechanics

Initial in vivo MRE studies of disc (Fig. 4d) demonstrated feasibility and repeatability^205,206^ but reported inconsistent trends with degeneration^207^: one study observed increasing stiffness with greater degeneration across participants with a range of Pfirrmann scores, while the other reported the opposite – decreasing stiffness with degeneration – which is more consistent with other studies^172,208^. These discrepancies may reflect methodological differences (e.g., actuator design, imaging sequence, inversion algorithm) that also contribute to wide variation in the reported healthy NP shear modulus values, ranging from ~6 to 26 kPa across studies^205,206,209^, though recent reports of shear wave speed have been relatively consistent, ~2–3 m/s which would correspond to ~1–10 kPa tissue modulus^199,208^. Notably, the range of reported in vivo shear moduli with MRE is well below reports from ex vivo mechanical testing with moduli in the range of tens to hundreds of kPa^128,210–213^.

This variability and discrepancy with ex vivo data is likely exacerbated by fundamental technical barriers to disc MRE, such as the small size of the disc and its relatively high stiffness causing wavelengths larger than the disc dimensions, and the bounded nature of the disc between vertebral bodies. In such cases where only a portion of a wavelength is captured by MRE throughout a tissue, current inversion algorithms tend to underestimate true stiffness, which is likely a primary source of discrepancy in reported in vivo disc mechanics with MRE. To address these limitations, methodological refinement is needed. Increasing vibration frequency can shorten the wavelength to improve property estimates and has shown better agreement in ex vivo disc specimens^214,215^, though higher frequencies exhibit greater attenuation and may not be achievable in vivo, especially for disc MRE where vibrations must be transmitted through several tissues to reach the disc.

Validation of MRE outcomes against expected mechanical properties is critical but also challenging. Since ex vivo mechanical studies introduce changes in tissue environment and boundary conditions that alter the apparent mechanical properties and are rarely performed in a similar mechanical regime to MRE (i.e., dynamic shear testing at a matched driving frequency), it is unlikely that such in vivo and ex vivo measurements could ever directly agree. One approach is to use MRE on ex vivo tissues for comparison with in vivo MRE^172,216^, which can allow for better matched comparisons and can also guide improvement in protocols.

### MRE – spinal muscle mechanics

In contrast to the disc, MRE is more developed for muscle applications. Early work in limb muscles showed muscle’s functional response to activities and with various pathologies^217–220^. Spinal muscle MRE feasibility was established in the 2010s^221,222^. Across studies, muscle stiffness is relatively consistent, with moduli of the paraspinal muscles typically in the 1.5–2.0 kPa range and psoas between 1.0 and 3.0 kPa^221,223,224^. Importantly, spinal muscle MRE is sensitive to physiological changes (Fig. 4e): stiffness increases with stretch and contraction, decreases after kinesio taping, and dynamically fluctuates after exercise, before returning to baseline^225–227^. This responsiveness demonstrates functional utility, suggesting that MRE can track interventions and rehabilitation outcomes in vivo.

Technical advances have improved spinal muscle MRE, including tailored imaging sequences^222^ and more comfortable actuators^223^. Still, reproducibility depends on muscle group and protocol, with paraspinal muscles generally yielding more consistent results than psoas, possibly due to the psoas location being more difficult to vibrate^228^. The aligned muscle fibers cause anisotropic (direction-dependent) tissue properties and traditional assumptions of tissue isotropy decrease the accuracy of material property calculations, which have been improved by advances in inversion approaches^229^. Yet, MRE evaluations remain limited by a lack of sufficient inversion algorithms to capture anisotropic material properties; improved algorithms will both reduce measurement uncertainty and improve interpretation of functional responses under loading^230–233^.

### MRE – outlook

MRE shows strong promise for advancing spine imaging by providing quantitative, noninvasive measures of mechanical function with a wide field of view to capture multiple tissues at once, sensitivity to deep tissues, and reduced operator dependence compared to USE. Additionally, MRE’s compatibility with other MRI approaches (e.g., T2 mapping, diffusion tensor imaging, loading MRI) will enhance mechanistic interpretations. Although consensus protocols are not yet mature, methodological refinements are rapidly progressing. There remains a need for mechanical actuator designs that maximize patient comfort while accommodating a wide range of body sizes. For muscle applications, acquisition protocols that can achieve high spatial resolution across the elongated spinal musculature that can be paired with improved inversion algorithms for evaluating anisotropic material properties will result in more accurate, reliable, and useful measures. In the disc, achieving accurate mechanical property estimates will require higher frequency vibration to reduce wavelengths across the tissue and likely advanced inversion algorithms uniquely tailored to disc geometry. Conventional mechanical actuators that vibrate deep tissues via surface application, and thus are susceptible to viscosity effects and other sources of attenuation may not be sufficient, and achieving necessarily high frequencies on the order of 1000 Hz may require unique and potentially semi-invasive approaches, such as with a needle driver^234^ or with acoustic radiation force excitation^235^. An important consideration for interpreting MRE mechanical properties in spinal tissues is that all measurements are performed in the small strain regime assuming linear viscoelastic material behavior, which may differ from large strain behavior experienced with physiological loading in vivo due to material nonlinearity^213^, though examples exist from other organs for how to scale such parameters for mechanical modeling purposes^236,237^.

We note that there are many challenges for achieving accurate mechanical properties of spinal tissues with MRE, the resulting outcomes are likely still useful especially for clinical applications where these mechanical measurements are used to infer structural integrity and health of the tissue. Future work aiming to establish spinal MRE in this context will benefit from larger and more diverse cohorts, studies in symptomatic populations, and longitudinal interventions to evaluate treatment effects. Dynamic applications, such as assessing tissue responses during loading or rehabilitation, could further expand functional insights and yield clinically relevant measures. Despite ongoing technical challenges, current developments underscore the feasibility of spinal MRE and highlight its potential to improve diagnosis, monitor treatment, and advance understanding of in vivo spine biomechanics.

## Discussion and conclusions

This review highlights extensive progress in the development of noninvasive imaging techniques for quantifying in vivo lumbar spinal tissue mechanics and mechanical properties, with each of radiography, ultrasound, ultrasound elastography, magnetic resonance imaging, and magnetic resonance elastography offering distinct advantages and limitations. Radiography remains foundational for bony assessment and indirect disc kinematics but provides limited insight into soft tissues. USI and USE offer portability, safety, and sensitivity to functional changes, particularly in lumbar muscles, though challenges with standardization, operator dependence, and penetration depth remain. MRI has provided the most comprehensive assessments of both disc and muscle structure and mechanics, including diurnal loading, postural changes, and exercise responses, but findings remain limited by the constraints of the MRI bore to allowing full range of motions, biological variability, small sample sizes, and technical heterogeneity. MRE has shown promise for quantifying muscle mechanics, with sufficient reproducibility to justify clinical translation, though disc MRE continues to face methodological barriers.

Despite these imaging advances, a critical gap remains: distinguishing age-related changes from pathological alterations associated with LBP. Both discs and muscles undergo degeneration with age, yet correlations with symptoms are inconsistent, and current imaging cannot reliably separate expected aging from pain-inducing pathology. Progress will require carefully designed studies that integrate functional measures, evaluate symptomatic populations, and account for age, sex, and spinal-level dependence. Moreover, clarifying the interplay between disc and muscle health, particularly whether degeneration in one accelerates dysfunction in the other, will be essential for identifying causal mechanisms and guiding therapeutic interventions.

Although this review focused on the lumbar spine and LBP, the same approaches are highly relevant to the cervical spine and neck pain. Neck pain is the second most common musculoskeletal disorder worldwide and ranks fifth on the Global Burden of Disease list of disabling conditions^35,36^. As with LBP, specific causes are often difficult to identify, making idiopathic neck pain a particularly impactful condition worldwide. Advances in in vivo assessment of spinal tissue mechanics hold promise for improving the diagnosis and treatment of idiopathic neck pain as well.

The next major opportunity lies in combining imaging-derived mechanical biomarkers with clinical and behavioral data using advanced computational approaches. Machine learning and artificial intelligence (AI) have the potential to integrate high-dimensional imaging outputs (e.g., strain maps, stiffness estimates, signal intensity patterns) with patient-specific demographic, clinical, and outcome data to improve diagnostic specificity, predict progression, and guide personalized treatment. Automated segmentation, strain quantification, and multimodal data fusion are already showing potential to overcome technical barriers and improve reproducibility. Ultimately, linking in vivo spinal mechanics with clinical outcomes through machine learning could transform spine care—moving from descriptive imaging toward predictive, mechanistically informed diagnosis and treatment.
